# Tislelizumab versus sorafenib as first-line treatment for advanced hepatocellular carcinoma in China: a cost-effectiveness analysis

**DOI:** 10.3389/fpubh.2024.1356244

**Published:** 2024-03-18

**Authors:** Zhiwei Zheng, Yuxuan Lin, Hongfu Cai

**Affiliations:** ^1^Department of Pharmacy, Cancer Hospital of Shantou University Medical College, Shantou, China; ^2^College of Pharmacy, University of Nottingham, Nottingham, United Kingdom; ^3^Tianjin University of Traditional Chinese Medicine, Tianjin, China; ^4^Department of Pharmacy, Fujian Medical University Union Hospital, Fuzhou, China

**Keywords:** cost-effectiveness analysis, partitioned survival model, tislelizumab, sorafenib, advanced hepatocellular carcinoma

## Abstract

**Objective:**

The goal of this study is to compare the cost-effectiveness of tislelizumab and sorafenib as first-line treatment for advanced hepatocellular carcinoma in China.

**Methods:**

A comprehensive cost-effectiveness analysis was undertaken within the framework of a partitioned survival model to accurately gage the incremental cost-effectiveness ratio (ICER) of tislelizumab compared to sorafenib. The model incorporated relevant clinical data and all survival rates were from RATIONALE-301 trials. The stability of the partitioned survival model was assessed by performing one-way and two-way sensitivity analyses.

**Results:**

The total cost incurred for the tislelizumab treatment was $16181.24, whereas the sorafenib was $14306.87. The tislelizumab regimen resulted in a significant increase of 0.18 quality-adjusted life years (QALYs) and an extra cost of $1874.37 as compared to chemotherapy. The ICER was $10413.17 per QALY, which was found to be below the willingness-to-pay (WTP) threshold of $37304.34/QALY. The results of the sensitivity analysis found that no fluctuations in any of the factors affected our results, even when these parameters fluctuated.

**Conclusion:**

Tislelizumab appears to be a cost-effective first-line treatment for advanced hepatocellular carcinoma when compared to sorafenib in China. These findings can inform decision-making processes regarding the selection of the most cost-effective treatment option for advanced hepatocellular carcinoma.

## 1 Introduction

Hepatocellular carcinoma (HCC) stands as the most prevalent and aggressive form of liver cancer on a global scale, presenting a persistent and formidable challenge in the field of oncology (1). According to the reliable and comprehensive data sourced from the GLOBOCAN database for the year 2022, it is projected that the worldwide incidence of HCC will witness a remarkable increase, reaching an alarming figure of 866,136 million cases. Out of this total, an estimated 367,657 million cases are expected to occur specifically in China. The mortality rate of HCC is projected to reach a distressing figure of 758,725 million cases worldwide. Notably, it is anticipated that approximately 316,544 million cases will occur specifically in China (2). Regrettably, it continues to maintain a prominent role as a major cause of mortality linked to cancer-related deaths worldwide. The prognosis for advanced HCC is generally poor, with a median survival time ranging from one to 3 years. Consequently, effective systemic therapies are crucial in improving patient outcomes (3). Currently, the most widely recommended initial systemic therapies for advanced HCC involve the utilization of single-agent multitargeted tyrosine kinase inhibitors (TKIs), with sorafenib being particularly prominent in this regard (4, 5). To date, novel targeted therapies and immunotherapeutic strategies are being developed and evaluated in clinical trials (6). Immunotherapy has recently emerged as a revolutionary treatment strategy for advanced HCC, harnessing the potent capabilities of the body’s immune system to specifically identify and eliminate malignant cells. This novel therapeutic approach holds great promise in combatting the formidable challenges posed by this aggressive form of liver cancer (7). Immune checkpoint inhibitors (ICIs) have revolutionized the treatment of advanced HCC by targeting inhibitory pathways that dampen the immune response. These inhibitors, such as programmed death-1 (PD-1) or programmed cell death 1 ligand 1 (PD-L1) inhibitors, have exhibited significant efficacy in a specific subset of HCC patients characterized by distinct molecular features (8). The introduction of the combination therapy comprising of atezolizumab and bevacizumab has emerged as a substantial breakthrough in the management of advanced HCC (9). Atezolizumab, as a PD-L1 inhibitor, exerts its mechanism of action by bolstering the immune system’s capability to identify and eliminate malignant cells. When administered alongside bevacizumab, an anti-vascular endothelial growth factor (VEGF) monoclonal antibody, this therapeutic combination exhibits enhanced efficacy in terms of both overall survival and disease progression, surpassing the effectiveness of sorafenib. The results of the IMbrave150 clinical trials have significantly transformed the treatment paradigm, underscoring the remarkable potential of ICIs in conjunction with antiangiogenic agents for HCC. This groundbreaking regimen offers a new approach to combatting advanced HCC, providing patients with improved survival outcomes and a glimmer of hope in their battle against this aggressive cancer (10).

In a recent RATIONALE-301 study, the effectiveness and safety of tislelizumab were evaluated as a therapeutic approach in comparison to sorafenib for patients diagnosed with advanced HCC. The study findings revealed that tislelizumab provided a significant overall survival benefit that was noninferior to sorafenib (11). This study made a noteworthy observation that tislelizumab demonstrated a significantly higher objective response rate when compared to sorafenib. This implies that a larger proportion of patients treated with tislelizumab experienced a reduction in tumor size or absence of tumor progression, thereby highlighting its potential as an effective therapeutic option for HCC. Furthermore, the responses observed with tislelizumab were found to be more long-lasting, suggesting that the positive effects of the treatment persisted for a longer duration when compared to sorafenib. Regarding safety, tislelizumab demonstrated a favorable safety profile compared to sorafenib, indicating that it may be well-tolerated by patients with a lower incidence of adverse events or treatment-related toxicities.

However, the increasing costs associated with immunotherapy medications have raised significant concerns within the healthcare community. Consequently, the field of oncology has seen a surge of interest in value-based healthcare, emphasizing the need for effective yet cost-effectiveness treatment options. Currently, there are relatively few cost-effectiveness analyses of PD-1 or PD-L1drugs in the treatment of HCC. Recently, Lang et al. conducted a comprehensive analysis utilizing a Markov state-transition model to assess the cost-effectiveness of different treatment approaches for HCC (12). The study was built upon the outcomes of the Phase 3 randomized CARES-310 clinical trial, which aimed to compare the efficacy of camrelizumab plus rivoceranib with sorafenib as a first-line therapy for unresectable HCC. The findings of this investigation revealed that camrelizumab plus rivoceranib exhibited superior cost-effectiveness compared to sorafenib when utilized as a first-line therapy for unresectable HCC in China.

Nevertheless, there remains a dearth of comprehensive evaluations regarding the cost-effectiveness of tislelizumab in comparison to sorafenib. Consequently, this study aims to provide an in-depth assessment of the cost-effectiveness of tislelizumab compared to sorafenib, thereby presenting vital information on the additional costs required to attain an additional unit of health benefit. The primary objective of our comprehensive study is to analyze the cost-effectiveness of implementing tislelizumab, an emerging immunotherapeutic agent, as a first-line treatment option for advanced HCC in China, in comparison to sorafenib, the current standard of care.

By conducting this thorough cost-effectiveness analysis, our study aims to bridge the existing research gap and contribute to the understanding of the economic value of utilizing tislelizumab based therapy for advanced gastric cancer. The outcomes of this analysis will not only offer valuable insights for clinicians and policymakers but will also facilitate informed decision-making regarding resource allocation and optimal management strategies for this devastating disease.

## 2 Methods

### 2.1 Partitioned survival model structure

**Figure 1:**
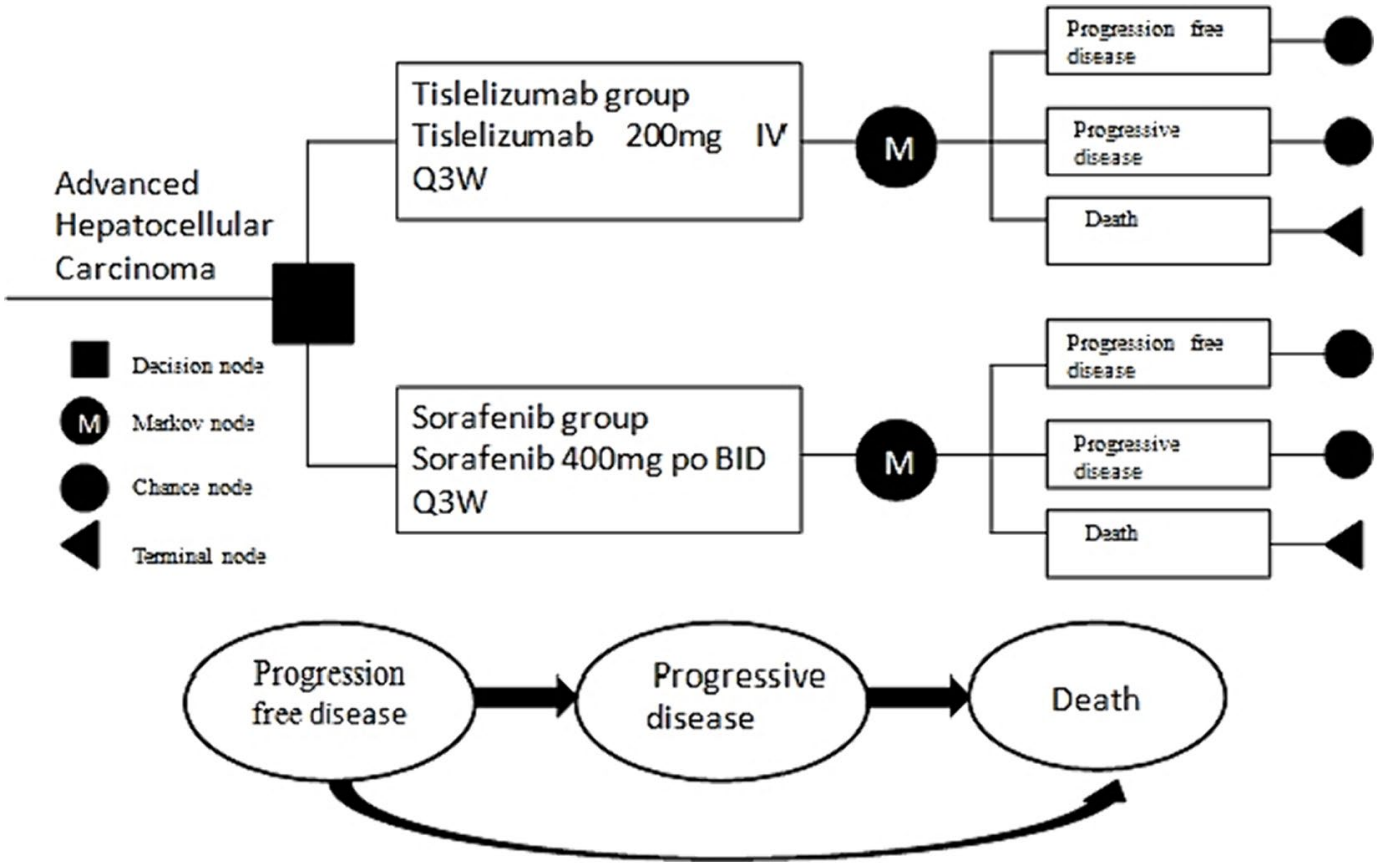
Partitioned survival model structure.

We have developed a partition survival model (PSM) to provide a comprehensive evaluation of economic and clinical outcomes with advanced HCC, which encompasses three distinctive and mutually exclusive states, specifically progression-free disease (PFD), progressive disease (PD), and death (Figure 1). All patients were assumed to initially enter the model in the PFD state, which represents their baseline health status. It was assumed that patients could either remain in their designated health state or transition to another health state in each cycle of the model. This assumption aligns with the dynamic nature of health conditions, where individuals may experience changes in their health status over time. By incorporating the possibility of transitioning between health states, the model accounts for the fluid nature of health and the potential for improvement or deterioration in patients’ health statuses over time. This dynamic aspect enhances the realism and applicability of the model in capturing the complex interplay between different health outcomes and their influence on patients’ overall well-being.

The model incorporates several significant direct healthcare costs, including expenses related to medication, managing adverse events, subsequent therapies, and best supportive treatment. In order to streamline the modeling procedure, Our survival model focuses on analyzing and comparing grade 3 or 4 adverse events with a frequency exceeding 10% in two separate groups.

We have established a simulation cycle period of 21 days for our PSM, which corresponds to the observed duration of the RATIONALE-301 clinical trial that took place over a span of 10 years. Additionally, we have taken into consideration a willingness-to-pay (WTP) threshold of $37304.34 per quality-adjusted life year (QALY), which is three times the national gross domestic product (GDP) in 2022 (13). The PSM was constructed utilized the TreeAge Pro 2011 software.

### 2.2 Population and treatment

The present study focuses on a population consisting primarily of patients who were consistent with the RATIONALE-301 trial. These patients were aged 18 years or older who had not received systemic therapy before and had histologically confirmed HCC.

From December 27, 2017 to October 2, 2019, a comprehensive study was conducted involving a total of 674 patients who were randomly assigned to receive different treatment regimens. Among these participants, 342 patients were allocated to the tislelizumab arm, while the remaining 332 patients were allocated to the sorafenib arm. In the tislelizumab cohort, patients were administered a dosage of 200 mg intravenously every 3 weeks, whereas those belonging to the sorafenib group received oral of 400 mg sorafenib twice daily. The treatment duration was extended until the occurrence of symptomatic deterioration linked to disease progression or the emergence of unacceptable toxic effects. In the tislelizumab arm, the median duration of treatment was reported as 4.1 months, varying between 0.6 and 50.4 months. Conversely, the sorafenib arm exhibited a slightly lower median treatment duration of 2.7 months, spanning from 0.0 to 49.0 months.

After disease progression, a subsequent systemic anticancer therapy was administered to 185 patients (54.1%) in the tislelizumab cohort and 199 patients (59.9%) in the sorafenib cohort. Following established guidelines and reports from the RATIONALE-301 clinical trial, the potential of lenvatinib or sintilimab plus bevacizumab as a subsequent anticancer therapy for the two treatment groups was considered, in order to conduct a comprehensive cost analysis. However, uncertainty surrounds the optimal choice for third-line therapy. As a result, it was hypothesized in our study that the best supportive treatment option would present as the most efficacious choice upon subsequent disease progression.

### 2.3 Data extraction and transition probabilities

Survival data from the treatment arm were meticulously obtained through the digitization of the survival curves derived from the RATIONALE-301 trial. This process was executed using the software of GetData Graph Digitizer. Subsequently, these curves were reconstructed using the R software to determine appropriate statistical distributions for modeling the survival curves. Our methodology for determining the optimal distribution involves a combination of visual inspection and minimum statistical criteria of the Akaike Information Criterion (AIC) and Bayesian Information Criterion (BIC). Ultimately, it was unequivocally determined that the log-logistic distribution provided the best fit for simulating the survival curves. The results of this comprehensive evaluation can be accessed in Supplementary Table S1, which provides in-depth details. Additionally, Supplementary Figure S1 visually represents the reconstructed distribution curves for each respective treatment group. In order to enhance the predictive of our model, we extend its effectiveness beyond the follow-up period. To address this, we employed the simulation of survival times utilizing the log-logistic distribution. This enabled us to calculate the survival function S (t) = 1/(1 + λt^γ^), where λ and γ are estimated parameters obtained from the R software. The corresponding values for these parameters are displayed in Table 1.

**Table 1:** Log-logistic survival estimates parameters.

Variable	Tislelizumab arm	Sorafenib arm
Log-logistic OS shape (γ)	1.339	1.568
Log-logistic OS scale (λ)	0.0225	0.0145
Log-logistic PFS shape (γ)	1.687	1.986
Log-logistic PFS scale (λ)	0.117	0.0694

OS, Overall Survival; PFS, Progression-Free Survival.

### 2.4 Cost data and utility data

The primary focus of this study was on the direct medical costs, which encompassed various aspects such as medication expenses, the treatment of serious adverse events, follow-up, subsequent therapy costs and costs associated with best supportive care. To obtain data on drug costs, we utilized the China Data Platform^1^ (14), while information regarding other cost factors was derived from relevant literature sources. All costs were converted to US dollars using the average annual exchange rate of 1 US dollar to 6.73 yuan in 2022 (15). Furthermore, sensitivity analyses were conducted on various cost variables to evaluate the impact of cost-related factors on the study outcomes.

To evaluate the quality of life associated with each health condition in this study, we utilized a utility values ranging from 0 to 1. This utility values represents the worst and best health states, respectively. Due to the unavailability of explicit utility value data from the RATIONALE-301 clinical trial, we sourced utility values from published literature. Moreover, our model accounted for the negative utility associated with adverse drug events. Table 2 presents the detailed data regarding cost and utility values.

**Table 2:** The parameters of PSM.

Parameters	Baseline	Range		Distribution	Source
	Value	Minimum	Maximum		
**TEAE rate of tislelizumab group [no. (%)]**					
AST level increased	78 (23.10)	–	–	Beta	Qin et al. (11)
ALT level increased	56 (16.60)	–	–	Beta	Qin et al. (11)
Blood bilirubin level increased	42 (12.40)	–	–	Beta	Qin et al. (11)
Rash	34 (10.10)	–	–	Beta	Qin et al. (11)
**TEAE rate of sorafenib group [No. (%)]**					
AST level increased	93 (28.70)	–	–	Beta	Qin et al. (11)
ALT level increased	81 (25.00)	–	–	Beta	Qin et al. (11)
Blood bilirubin level increased	67 (20.70)	–	–	Beta	Qin et al. (11)
Rash	54 (16.70)	–	–	Beta	Qin et al. (11)
**Drug costs ($)**					
Tislelizumab (100 mg)	204.68	153.51	255.85	Gamma	Yao (14)
Sorafenib (200 mg)	2.30	1.73	2.88	Gamma	Yao (14)
**Cost of TEAE per cycle ($)**					
AST level increased	56.54	42.41	70.68	Gamma	Li and Wan (16)
ALT level increased	56.54	42.41	70.68	Gamma	Li and Wan (16)
Blood bilirubin level increased	77.28	57.96	96.60	Gamma	Zhou et al. (17)
Rash	42.15	31.61	52.69	Gamma	Zhou et al. (18)
Subsequent systemic therapy in tislelizumab group per cycle ($)	272.68	204.51	340.85	Gamma	Shu et al. (19)
Subsequent systemic therapy in sorafenib group per cycle ($)	330.09	247.57	412.61	Gamma	Shu et al. (19)
Best supportive care ($)	265.08	198.75	331.35	Gamma	Zhao et al. (20)
Follow-up cost per cycle ($)	59.20	44.40	74.00	Gamma	Xu et al. (21)
**Utility**					
Progression-free disease	0.76	0.57	0.95	Beta	Li et al. (22)
Progressive disease	0.68	0.51	0.85	Beta	Li et al. (22)
Rash	−0.16	−0.12	−0.2	Beta	Li et al. (23)
Discount rate	5%	0	8%	Beta	Yue et al. (24)

TEAE, Treatment-emergent adverse event; AST, aspartate aminotransferase; ALT, alanine aminotransferase.

### 2.5 Sensitivity analysis

The stability of the model was assessed by performing one-way and two-way sensitivity analyses. In the one-way analyses, each input parameter was subject to adjustment by ±25%, allowing for an in-depth examination of how changes in these parameters might influence the ICER. Notably, the discount rate was varied between 0 and 8% in line with the recommendations of the Chinese guidelines for pharmacoeconomic evaluation. In our study, we conducted a two-way sensitivity analysis to assess the collective impact of simultaneously altering the cost of subsequent treatment values and other parameters on model outcomes. This analysis was conducted as our earlier one-way sensitivity analyses indicated a significant influence of the cost of subsequent treatment on ICER values. The results of this analysis were visually depicted using a tornado diagram, showcasing the relative influence of each parameter on the ICER.

Additionally, to comprehensively assess the uncertainty associated with the ICER, a probabilistic sensitivity analysis was performed. This analysis involved the execution of 1,000 Monte Carlo simulations, allowing for a more extensive exploration of various probabilistic scenarios. The results of this analysis were summarized using acceptance curves, which offer a visual depiction of the likelihood of achieving cost-effectiveness at different WTP thresholds. By employing acceptance curves, we were able to graphically represent the probability of the intervention being considered cost-effective across a range of WTP values.

## 3 Results

### 3.1 The results of base case analysis

The total cost for the tislelizumab was $16181.24, while the sorafenib incurred a total cost of $14306.87. It was observed that the tislelizumab experienced a statistically significant benefit of 1.24 QALYs, whereas the sorafenib gained 1.06 QALYs. These findings indicate that the tislelizumab regimen resulted in a substantial increase of 0.18 quality-adjusted life years compared to sorafenib. The additional cost associated with the tislelizumab treatment amounted to $1874.37 compared to the sorafenib. Consequently, the calculated ICER was $10413.17/QALY. The determined ICER value from this analysis was found to be below the Chinese WTP threshold of $37304.34/QALY. This implies that the use of tislelizumab as a treatment regimen for patients with advanced HCC is considered cost-effective when compared to the sorafenib group. Table 3 provides a comprehensive overview of all the relevant data in this analysis.

**Table 3:** The base case results.

Parameters	Tslelizumab group	Sorafenib group
Cost ($)	16181.24	14306.87
QALYs	1.24	1.06
Incremental cost ($)	1874.37	NA
Incremental QALY	0.18	NA
ICER ($/QALY)	10413.17	NA

ICER, Incremental cost–effectiveness ratio; QALY, Quality-adjusted life year; NA, Not applicable.

### 3.2 The results of sensitivity analysis

**Figure 2:**
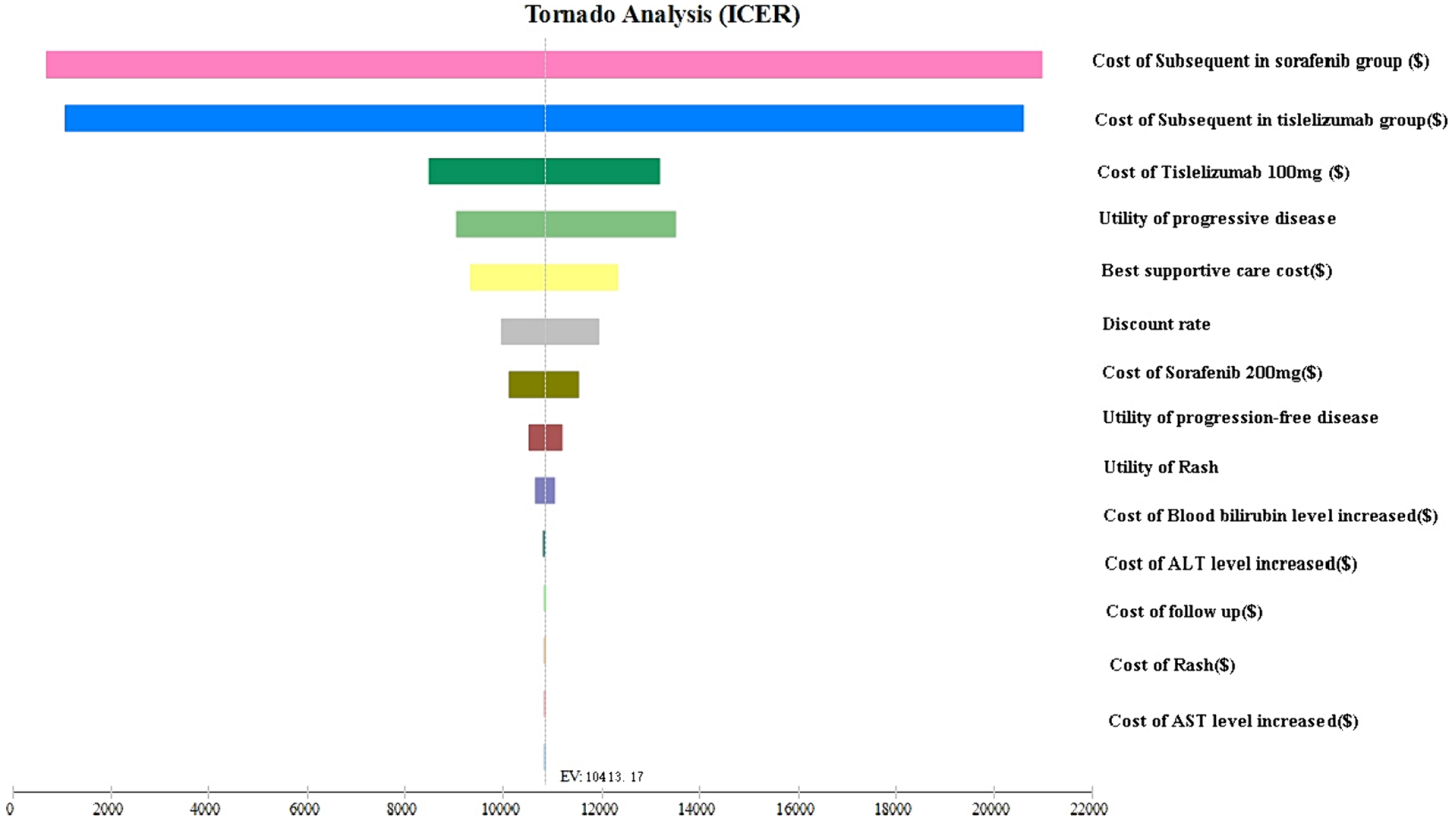
The tornado plot of the one-way sensitivity analysis.

The tornado plot displayed in Figure 2 presents the findings of the sensitivity analysis. Notably, the parameter with the greatest influence on the ICER was the cost of subsequent treatment. Based on our hypothesis regarding the varying costs of second-line regimens for different groups, we discovered that as the cost of these treatments increased, the ICER also increased. This finding suggests that higher costs were associated with a greater impact on ICER outcomes. However, it is important to note that despite this increase, the ICER did not surpass the acceptable threshold determined by the WTP value.

Factors such as the cost of tislelizumab, the cost of sorafenib, the utility value of disease progression, the utility value of progression-free disease, and the cost of best supportive care also exerted some impact on the ICER. The dynamic interaction between these variables reveals a discernible pattern wherein the escalation in drug prices invariably leads to a concomitant rise in the ICER associated with our intended outcome. This intricate relationship underscores the pivotal role played by cost in influencing the ICER value. Additionally, the utility of PD and PFS were influences on the model ICER result, The utility value serves as a measure of the health-related quality of life associated with different health conditions. A lower utility value indicates a poorer health status, thereby implying a greater impact of ICER on the outcome. Conversely, a higher utility value suggests a better health status, leading to a lower ICER value associated with the outcome. It is worth emphasizing that altering these parameters within a range of ±25% did not significantly alter the results of the analysis. Furthermore, the ICER values consistently remained below twice the GDP, indicating that they were well below the predetermined threshold of WTP. This observation underscores the robustness of the model and confirms the stability of the results, even when these parameters fluctuate.

Two-way sensitivity analyses consistently indicated that the tislelizumab group remained a cost-effective treatment option, regardless of variations in the cost of subsequent treatment in both the sorafenib group and the tislelizumab group (Supplementary Figure S2).

**Figure 3:**
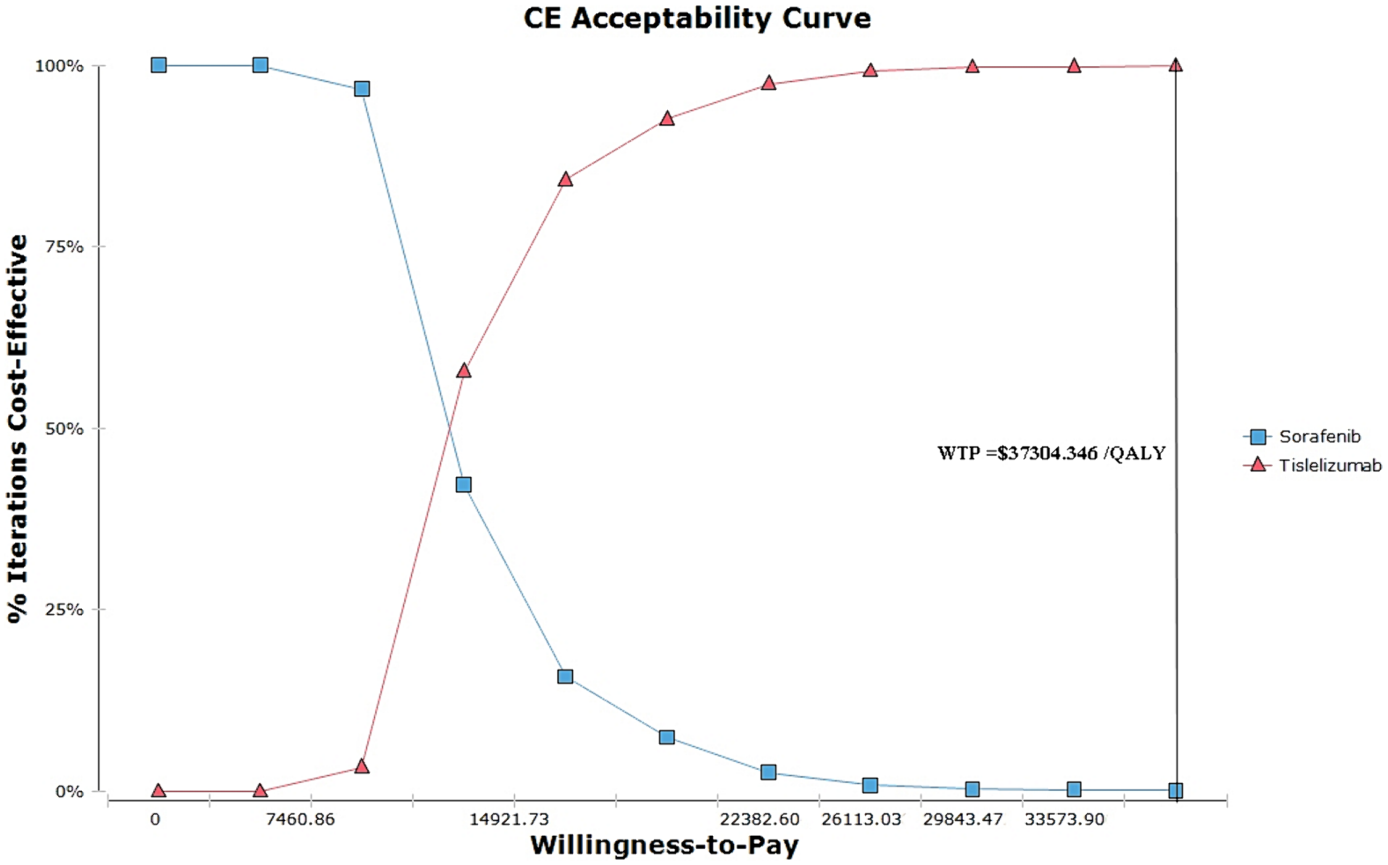
The acceptability curves of the Monte Carlo simulations.

Figure 3 depicts the acceptability curves illustrating the outcomes of the Monte Carlo simulations. Notably, at a WTP threshold of $37304.346 per QALY, the probability of considering the tislelizumab regimen as a more cost-effective option is exceedingly high, at approximately 99.99% compared to the sorafenib group. When the ICER values hover around 1 times the GDP, specifically at 14921.736 per QALY, the tislelizumab regimen is deemed to be 84.20% more cost-effective than the sorafenib group.

**Figure 4:**
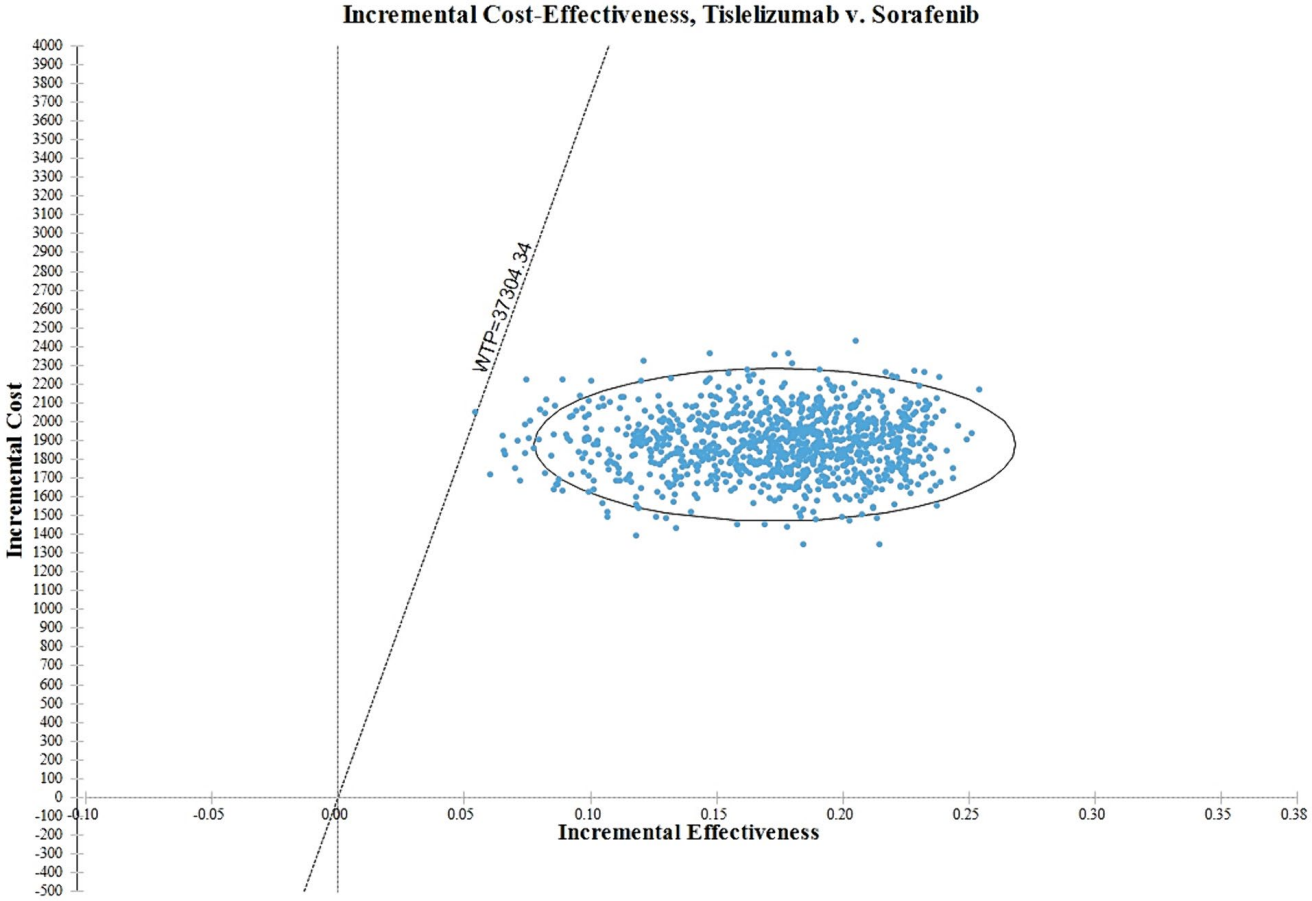
The ICER plane of tislelizumab versus sorafenib for patients with HCC.

In Figure 4, the ICER plane is divided into quadrants, and the distribution of the 1,000 bootstrap replicates of the ICER is depicted. According to the findings, interventions falling in the North-East quadrant of the cost-effectiveness plane, along the linear ICER line, are perceived as cost-effective. This is attributed to their ability to generate net health benefits, indicating that the tislelizumab regimen is considered more cost-effective in comparison to the sorafenib group.

## 4 Discussion

Cancer constitutes a substantial public health concern and serves as a significant contributor to the global disease burden (25). According to the most recent data released by the World Health Organization in 2020, cancer has emerged as the leading cause of premature mortality in a significant proportion of nations worldwide (26). In China, in particular, cancer has maintained its position as the leading cause of death since 2010, with a progressively increasing incidence, mortality rate, and burden (27). In the year 2020, China is projected to witness a staggering number of cancer-related fatalities, reaching 2,397,772 individuals, with a mortality rate of 170.80 per 100,000 population (28). Lung, liver, and stomach cancers persist as the leading causes of mortality in China (29). These three forms of cancer consistently surface as the primary contributors to the overwhelming death toll within the nation. This alarming statistic underscores the pressing urgency to address the detrimental impact of cancer within the country.

The most recent advancements in immunotherapy have yielded encouraging outcomes in various cancer types, particularly in advanced HCC (30). Notably, the utilization of the PD-1 inhibitor tislelizumab has demonstrated substantial effectiveness in treating advanced HCC, predominantly by stimulating an anti-tumoral immune response (11). The RATIONALE-301 trial has effectively accomplished its primary objective of demonstrating the noninferiority in OS between single-agent tislelizumab and sorafenib when used as first-line therapy in patients afflicted with advanced HCC. Furthermore, tislelizumab showed superior objective response rates (ORR) and more enduring treatment responses compared to sorafenib. These findings indicate a potential for long-term survival advantages in patients receiving tislelizumab treatment. Although this therapeutic intervention has demonstrated impressive effectiveness in clinical trials, the potential financial implications of its implementation remain uncertain. It is crucial to ascertain whether the additional expenses associated with this therapy outweigh its potential benefits. Therefore, it is crucial to undertake an extensive economic evaluation of pharmaceutical drugs. Consequently, the principal aim of this investigation is to comprehensively evaluate the cost-effectiveness of tislelizumab in comparison to sorafenib as a primary therapeutic approach for advanced HCC.

According to the results obtained from our comprehensive research, the therapeutic intervention involving tislelizumab for the advanced HCC has exhibited a highly favorable ICER of $10413.17 per QALY gained compared to the conventional treatment approach using sorafenib. This ICER significantly falls below the widely accepted threshold of WTP of $37304.346 per QALY, which demonstrates tislelizumab was cost-effective compared to sorafenib in China.

In our study, the cost of subsequent treatment was identified as the primary factor influencing the results of the ICER. Specifically, the costs associated with subsequent treatment and drugs exhibited a direct impact on the overall cost estimated by our model. Consequently, as the costs of subsequent treatment and drugs decreased, the estimated cost by our model also decreased, leading to a decrease in ICER values. Conversely, an increase in the costs resulted in higher ICER values.

However, when we conducted a sensitivity analysis by adjusting all parameters within a range of ±25%, we found that the maximum ICER values did not exceed the threshold value established as the WTP threshold. This indicates the robustness and reliability of our findings, as even substantial variations in the input parameters did not yield ICER values surpassing the predetermined threshold. Two-way sensitivity analyses consistently demonstrated that the tislelizumab group remained a highly cost-effective treatment option, irrespective of variations in the cost of subsequent treatment in both the sorafenib group and the tislelizumab group. These sensitivity analyses were conducted to evaluate the robustness of the cost-effectiveness results and assess the impact of varying factors on the economic outcomes of different treatment options.

Throughout the analyses, it was consistently observed that the cost-effectiveness of tislelizumab as a treatment for the targeted condition was not significantly influenced by fluctuations in the costs associated with subsequent therapies for both the sorafenib group and the tislelizumab group. This finding suggests that tislelizumab provides consistent value for its cost, regardless of the expenses incurred in follow-up treatments for patients in either group.

This information holds particular significance from an economic standpoint as it indicates that the favorable cost-effectiveness profile of tislelizumab remains steadfast and unaffected by fluctuations in subsequent treatment costs. This further suggests that the initiation of tislelizumab therapy results in long-term cost savings, as compared to the alternative treatment option of sorafenib, even when the costs of follow-up therapies vary.

The results of the two-way sensitivity analyses provide robust evidence supporting the superior cost-effectiveness of tislelizumab, reinforcing its position as a highly viable and economically advantageous treatment option. This information can serve as a valuable resource for healthcare decision-makers, enabling them to make informed choices regarding the allocation of healthcare resources and prioritize the adoption of tislelizumab in clinical practice.

When it comes to determining the cost-effectiveness threshold, several key factors come into play, including the concept of WTP per QALY and a nation’s GDP *per capita* (31). These considerations hold significant importance in shaping the threshold, ensuring that valuable healthcare resources are allocated efficiently. WTP per QALY serves as a fundamental concept in evaluating the economic value of healthcare interventions. It embodies the idea that individuals are willing to allocate a specific monetary value in exchange for gaining one additional year of life in perfect health or a significant improvement in their quality of life. By quantifying this willingness to pay, decision-makers can better understand the relative worth of different healthcare interventions and allocate limited resources judiciously. GDP *per capita*, on the other hand, provides crucial insight into a nation’s overall economic capability and available resources for healthcare spending. As an indicator of a country’s economic performance and average income, GDP *per capita* offers a benchmark for establishing the cost-effectiveness threshold. The World Health Organization (WHO) recommends that national cost-effectiveness thresholds typically fall within the range of one-to-three times a country’s GDP *per capita* (32).

In this study, we adopted a threshold for the WTP of 3 times the GDP, equating to $37304.346 per QALY, in line with the Chinese Pharmacoeconomics Guidelines. Our analysis focused on the therapeutic intervention of tislelizumab for advanced HCC, comparing it to the standard treatment option of sorafenib. The ICER for tislelizumab was estimated to be $10413.17 per QALY gained compared to sorafenib. Notably, this ICER value is significantly below the widely accepted WTP threshold of $37304.346 per QALY. This finding indicates that the use of tislelizumab as a first-line treatment modality for advanced HCC has the potential to be considered cost-effective. Even in the current situation where some researchers and scholars are proposing to set the WTP at 1.5 times GDP (33), our present study is suggesting that the treatment of tislelizumab for advanced HCC is cost-effective in China.

There is currently a lack of reported cost-effectiveness analyses specifically examining first-line treatment with tislelizumab in advanced HCC. However, studies have been conducted on the cost-effectiveness of other ICIs in advanced HCC. For instance, Gong et al. provides a cost-effectiveness analysis of sorafenib, lenvatinib, atezolizumab combined with bevacizumab, and sintilimab combined with bevacizumab. The findings of these regimens were not cost-effective at a WTP threshold of US$36600 per QALY (34). In a similar vein, Tseng et al. conducted a cost-effectiveness of atezolizumab plus bevacizumab versus sorafenib treatment in Taiwan (35). The ICER was determined to be USD 75192/QALY, which fell below the predefined WTP threshold in Taiwan. Consequently, the combined treatment of atezolizumab plus bevacizumab was deemed cost-effective compared with the current first-line option of sorafenib for unresectable HCC in Taiwan. In addition, an intriguing comparative cost-effectiveness analysis was conducted to evaluate the economic implications of pembrolizumab compared to placebo administered as a second-line therapeutic regimen for patients afflicted with HCC from the perspective of US payers. The findings of this investigation unequivocally indicate that, given its current price point, pembrolizumab fails to exhibit cost-effectiveness as a second-line treatment for HCC in the United States (36).

It is crucial to acknowledge the limitations inherent in our study. Firstly, it is important to note that our data were sourced from clinical trials. While we employed robust research methodologies and utilized extensive data sources for our cost-effectiveness analyses, it is imperative to continuously monitor and update these findings as new evidence emerges and as costs and efficacy evolve over time. Additionally, further research is warranted to investigate the long-term efficacy and cost-effectiveness of our intervention in different patient subgroups, as well as the potential synergistic effects it may have when combined with other treatment modalities. Moreover, it is essential to recognize that our study made certain assumptions regarding the cost of second-line therapy after disease progression. However, it is essential to consider that in reality, the choice of subsequent treatment regimen will vary based on the individual circumstances of each patient. Fortunately, the results of our one-way sensitivity analyses provide reassurance, as they consistently demonstrate that the ICER values remain below the WTP threshold, even when altering the estimated range of subsequent treatments. Finally, one limitation of our study is the exclusion of grade 1 or 2 adverse events from our analysis. We made the assumption that their impact on clinical outcomes would be minimal. While this approach allowed for a concise modeling process, it may not fully capture the comprehensive impact of treatment-related toxicity on patient prognosis. Nonetheless, it is encouraging that the results of our sensitivity analyses demonstrate that even when considering grade 3 or higher adverse events, the variability range does not alter our overall conclusions.

## 5 Conclusion

Tislelizumab is a PD-1 antibody immunotherapy drug, while sorafenib is a targeted therapy drug. Although the two drugs have different mechanisms for treating hepatocellular carcinoma, both are equitably available in healthcare facilities in China. Although tislelizumab is currently only reimbursed under China’s health insurance for the treatment of HCC that has undergone at least one systemic therapy. However, tislelizumab holds promise as a cost-effective first-line treatment option for advanced HCC in comparison to sorafenib. Efforts must be made to ensure its coverage and accessibility in the Chinese healthcare system. Improving reimbursement policies providing adequate training for healthcare professionals are crucial steps toward optimizing treatment options for patients with advanced HCC.

## Data availability statement

The original contributions presented in the study are included in the article/Supplementary material, further inquiries can be directed to the corresponding author.

## Author contributions

ZZ: Investigation, Writing – original draft, Writing – review & editing. YL: Data curation, Formal analysis, Validation, Writing – original draft. HC: Resources, Supervision, Visualization, Writing – original draft.

## Footnotes

^1^ https://data.yaozh.com/
