# Supplementary material for: Tislelizumab versus sorafenib as first-line treatment for advanced hepatocellular carcinoma in China: a cost-effectiveness analysis
Source: Front Public Health. 2024 Mar 18;12:1356244. doi: 10.3389/fpubh.2024.1356244 (PMC10982498; doi:10.3389/fpubh.2024.1356244)
Supplement: Supplementary file 1 [file Data_Sheet_1.docx]

**Supplementary Table 1. Comparison of survival models**

|  | AIC | | BIC | |
| --- | --- | --- | --- | --- |
|  | Tislelizumab group | Sorafenib group | Tislelizumab group | Sorafenib group |
| PFS |  |  |  |  |
| Weibull | 1707.367 | 1315.120 | 1715.036 | 1322.731 |
| **Log-logistic** | **1552.726** | **1237.518** | **1560.395** | **1245.128** |
| Log-normal | 1568.844 | 1229.133 | 1576.514 | 1236.743 |
| Gompertz | 1609.812 | 1317.824 | 1647.482 | 1325.435 |
| Exponential | 1730.965 | 1319.332 | 1734.799 | 1323.137 |
| Gamma | 1727.645 | 1301.709 | 1735.315 | 1309.320 |
| OS |  |  |  |  |
| Weibull | 2083.478 | 2069.473 | 2091.148 | 2077.083 |
| **Log-logistic** | **2048.809** | **2034.358** | **2056.478** | **2041.968** |
| Log-normal | 2057.881 | 2042.079 | 2065.551 | 2049.689 |
| Gompertz | 2073.734 | 2071.831 | 2081.404 | 2079.441 |
| Exponential | 2081.582 | 2070.912 | 2085.417 | 2074.717 |
| Gamma | 2083.260 | 2065.082 | 2090.930 | 2072.692 |

AIC: Akaike information criterion; BIC: Bayesian Information Criterion; OS: Overall survival; PFS: Progression-free survival;


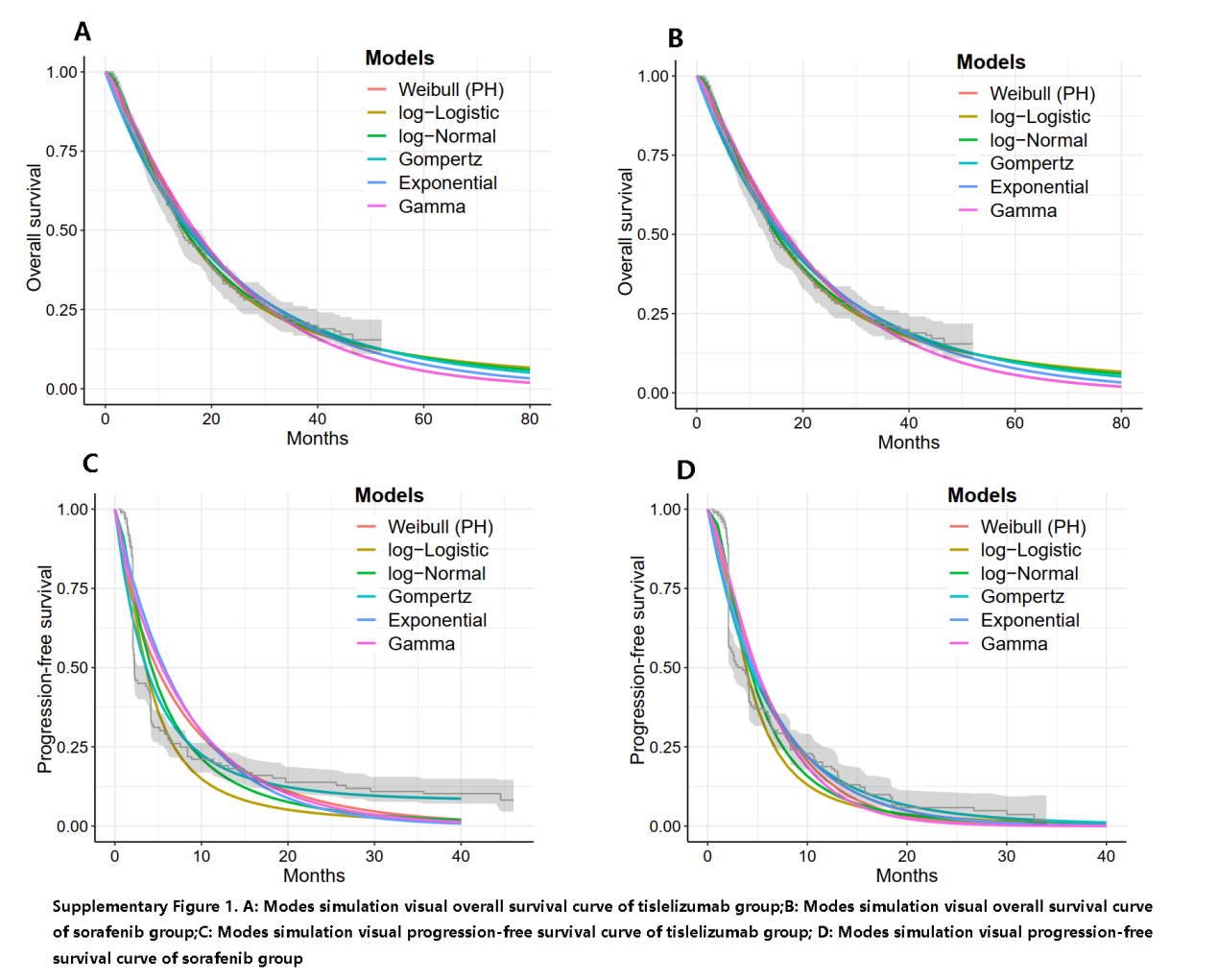


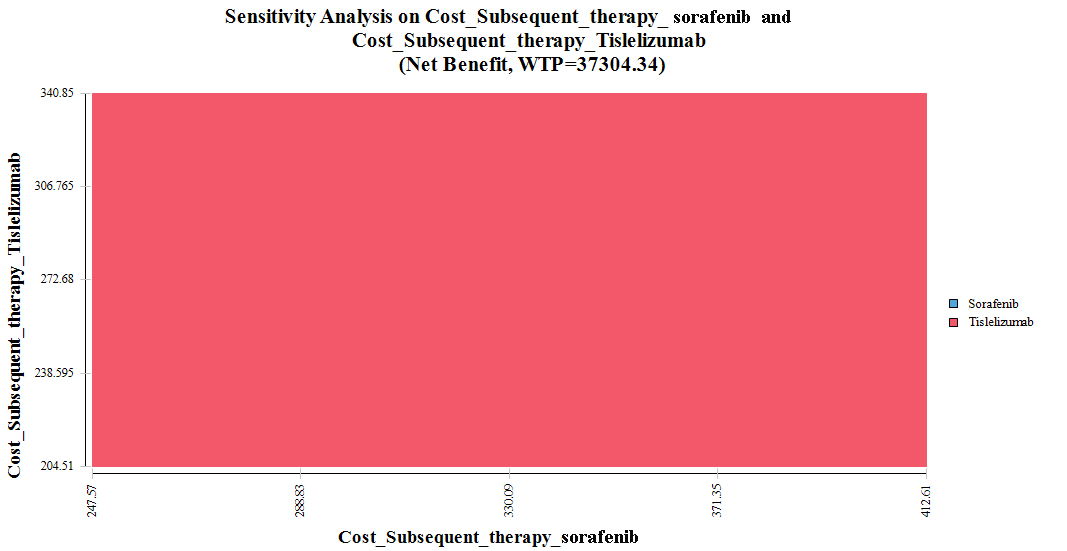


Supplementary Figure 2 The result two-way sensitivity analyses
